# Supplementary material for: Metabolic response to point mutations reveals principles of modulation of in vivo enzyme activity and phenotype
Source: Mol Syst Biol. 2021 Jun 27;17(6):e10200. doi: 10.15252/msb.202110200 (PMC8236904; doi:10.15252/msb.202110200)
Supplement: Supplementary file 1 — Expanded View Figures PDF [file MSB-17-e10200-s001.pdf]

Expanded View Figures

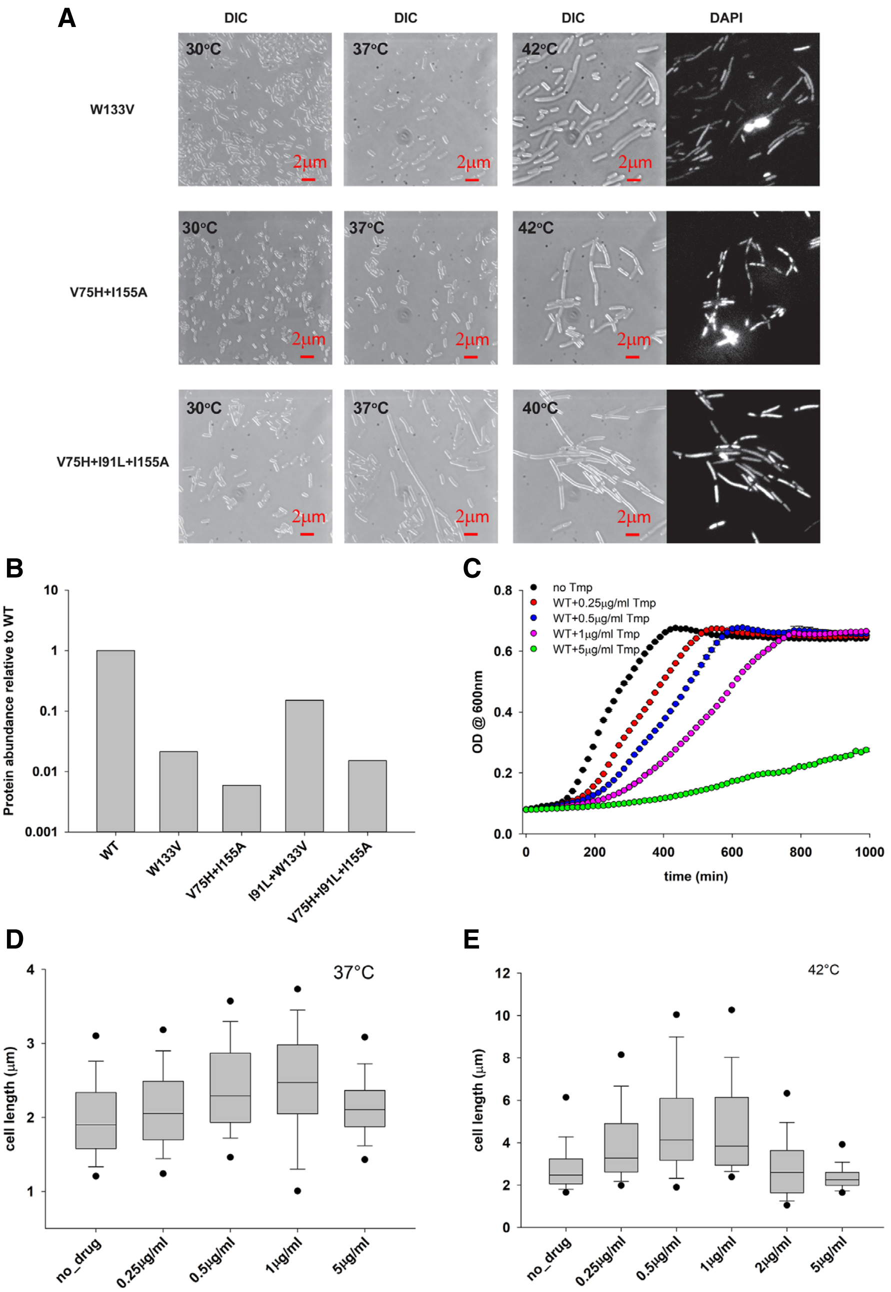

Figure EV1. Filamentation of mutant DHFR strains and effect of trimethoprim on filamentation.

A Destabilizing mutations in DHFR induce filamentous phenotype. Live cell DIC images with DAPI nucleoid staining of W133V, V75H+I155A, and V75H+I91L+I155A DHFR *E. coli* MG1655 strains. Prior to microscopy, cells were grown at 30, 37, and 42°C (V75H+I91L+I155A was grown at 40°C) in amino acid supplemented M9 medium for 4 h (see Materials and Methods).

B Intracellular abundance of WT and mutant DHFRs measured by Western blot. WT, W133V, and V75H+I155A were grown for 4 h at 42°C, while I91L+W133V and V75H+I91L+I155A strains were grown for 4 h at 40°C in amino acid supplemented M9 medium before being harvested. The data are also reported in Bershtein *et al* (2015a).

C The effect of WT DHFR inhibition by trimethoprim (Tmp) on growth. WT DHFR cells were grown at 42°C in amino acid M9 medium, and their growth was monitored by OD at 600nm. The data were fit to a 4-parameter Gompertz equation as described in Bhattacharyya *et al* (2017) to derive growth parameters.

D, E Distribution of cell length of WT *E. coli* as a function of Tmp concentration when grown in amino acid supplemented M9 medium at (D) 37°C and (E) 42°C. Concentrations of Tmp slightly below or near the MIC (1 μg/ml) result in maximum filamentation, while the effect dies down at higher concentrations. Filamentation is much more pronounced at 42°C than at 37°C. The central band in the boxplots represents the median of the distribution, the box ends represent the 25th and 75th percentiles, and the whiskers represent the 10th and 90th percentiles, while the dots represent the 5th and 95th percentiles. Data were usually obtained from 2-3 biological replicates. The number of cells used to derive the boxplot distributions in the different panels ranges usually between 200 and 500.

 © 2021 The Authors

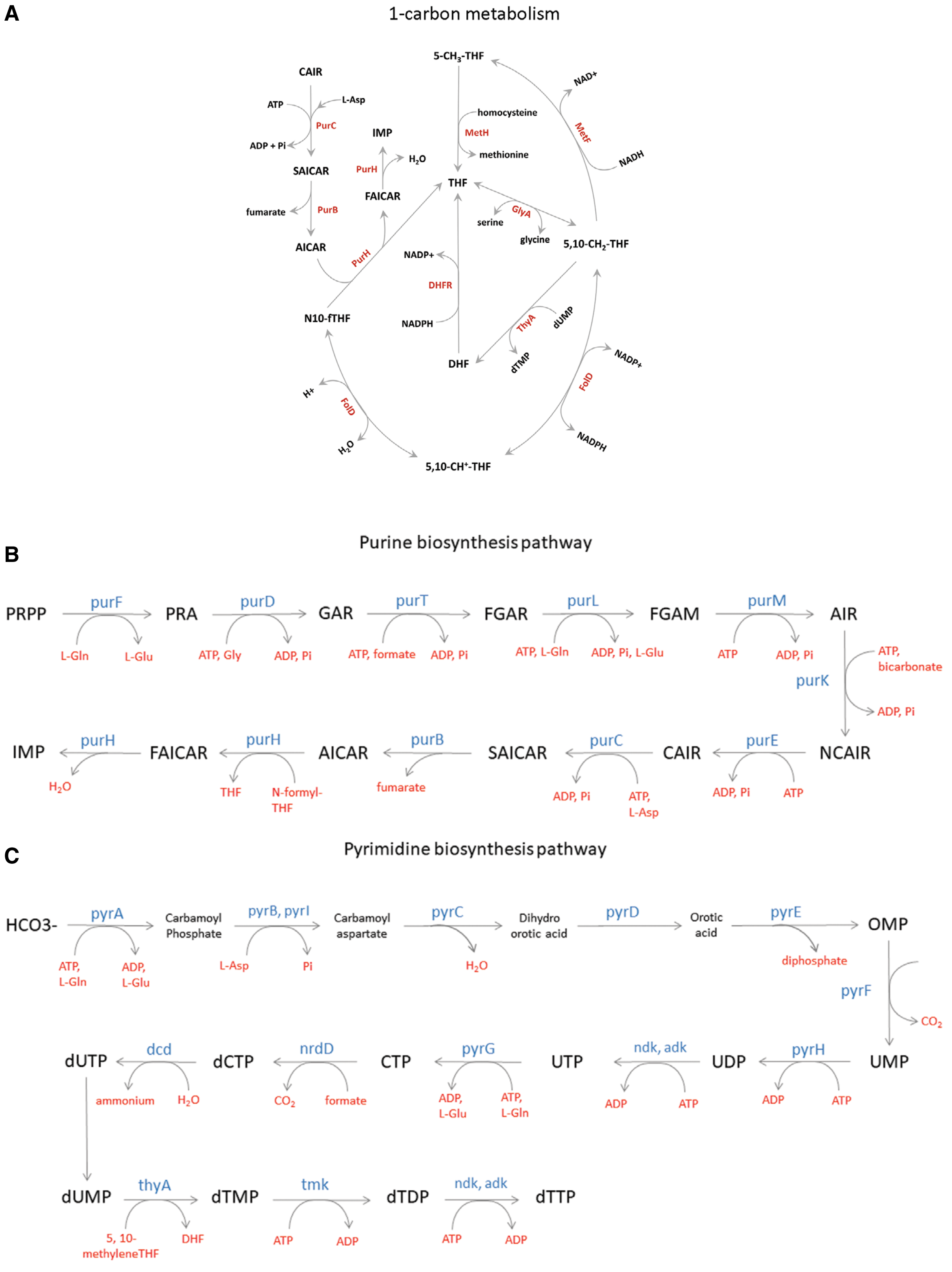

Figure EV2. Pathways that use up product of DHFR activity.

A–C Schematic representation of (A) 1-carbon metabolism pathway (adapted from Bhattacharyya *et al* (2016)), (B) *de novo* purine biosynthesis pathway, and (C) *de novo* pyrimidine biosynthesis pathway.

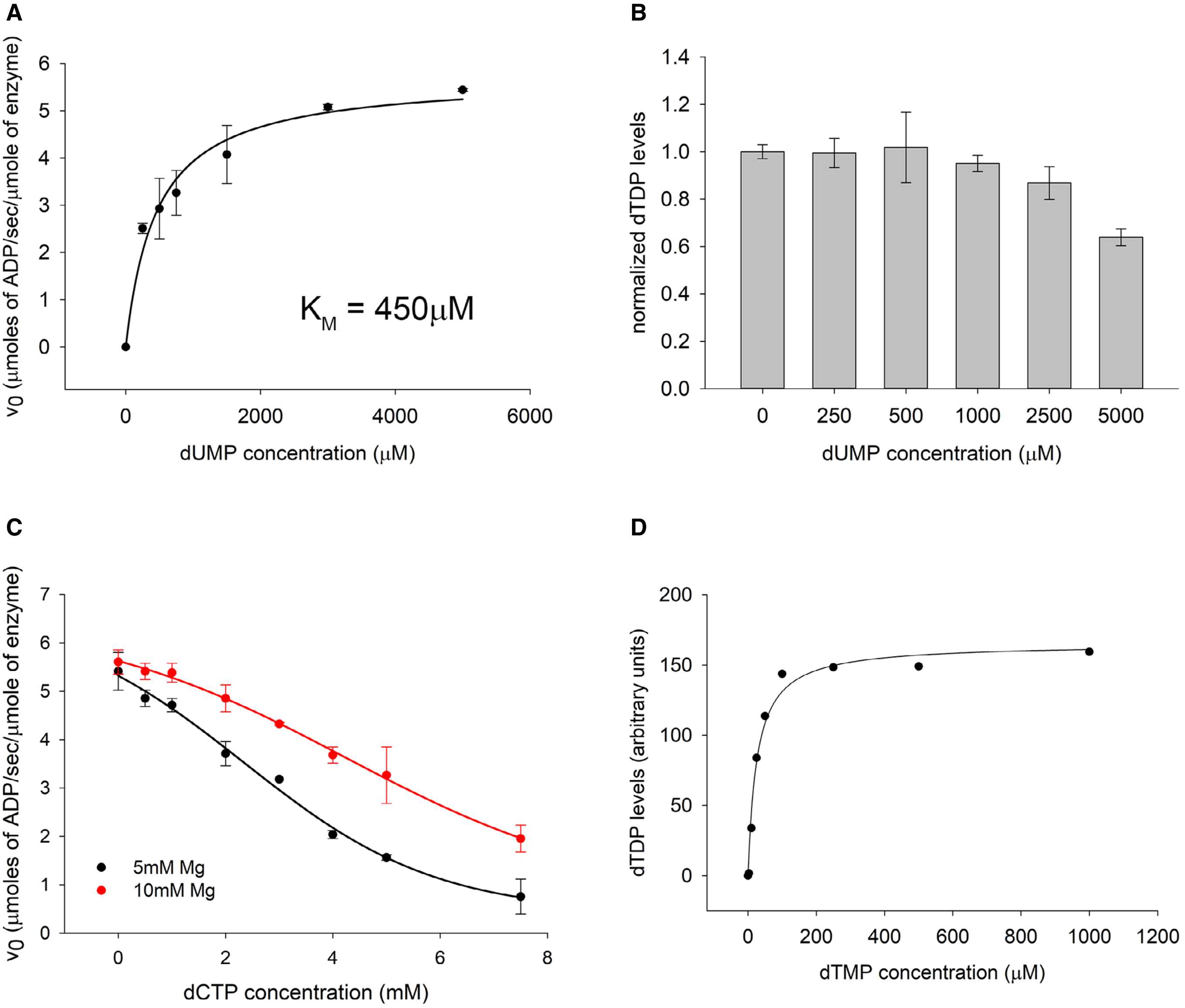

Figure EV3. Effect of dUMP and dCTP on the *in vitro* activity assay of thymidylate kinase (Tmk).

A Activity assay of purified Tmk enzyme using dUMP as substrate. ATP concentration is kept saturating at 1 mM. The K_M for dUMP is 450 µM, compared to 13 µM for dTMP.

B Activity assay of Tmk was carried out in the presence of 100 mM dTMP and 1 mM ATP and varying concentration of the inhibitor dUMP. dTDP levels were measured by HPLC followed by mass spectrometry. The data were fitted with a 4-parameter sigmoid curve to obtain an apparent K_I of 3.9 mM for dUMP.

C Activity assay of Tmk was carried out in the presence of 100 mM ATP and 1 mM dTMP and varying concentration of dCTP. ADP levels were measured using a NADH-based coupled spectrophotometric assay. The red and black points indicate data acquired under different concentrations of Mg^{2+}. The data were fitted with a 4-parameter sigmoid curve to obtain apparent K_I of 2.3 and 4.2 mM at 5 and 10 mM Mg^{2+} concentrations, respectively. For panels A to C, the error bars represent SD of three technical replicates.

D Activity assay of purified Tmk as a function of dTMP concentration in the presence of 5 mM dUMP and 2.5 mM dCTP as inhibitors. ATP concentration was kept saturating at 1 mM. The dTDP levels were monitored using HPLC followed by mass spectrometry. Even in the presence of inhibitors, the activity data here conform to MM kinetics.

 © 2021 The Authors

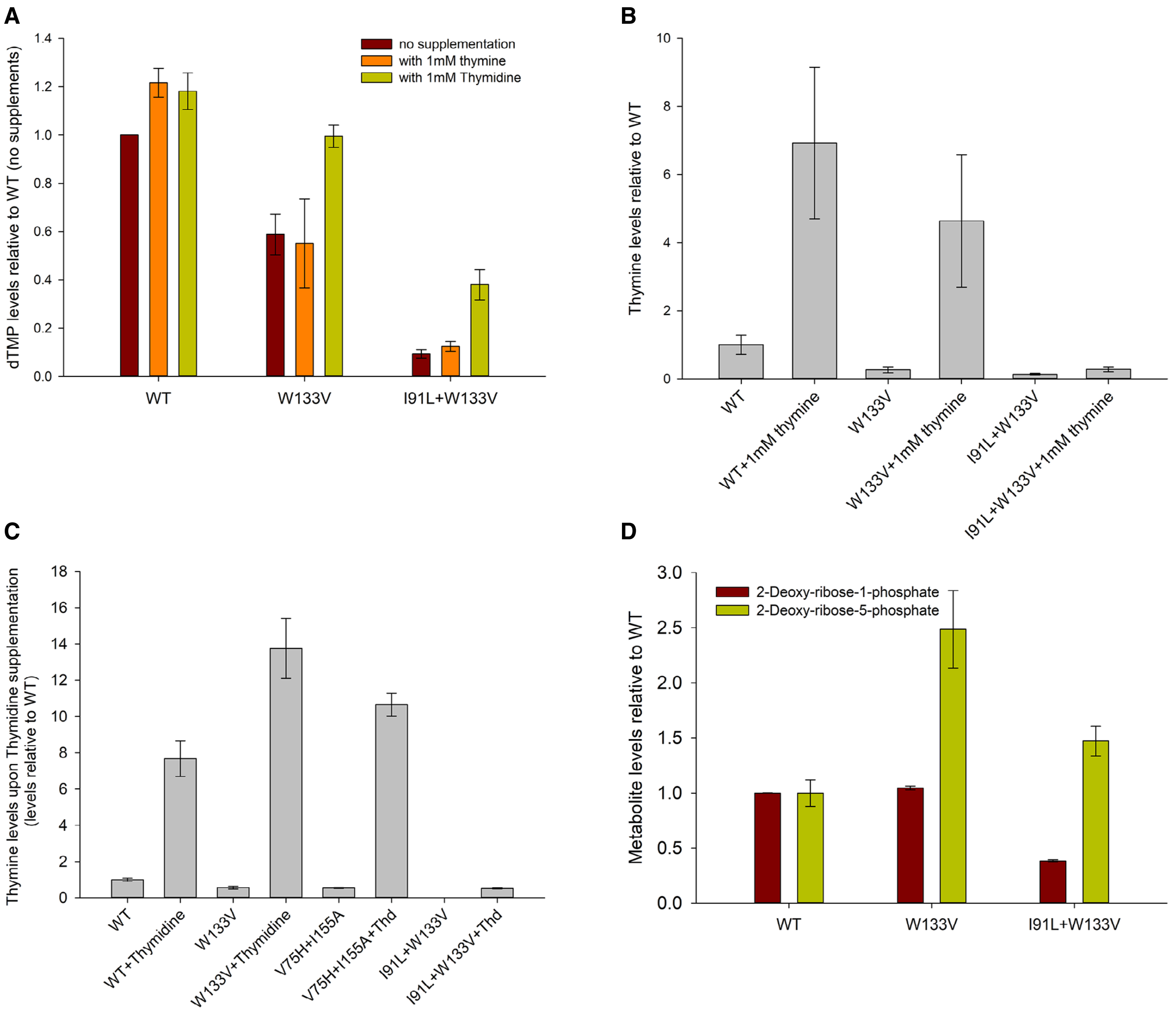

Figure EV4. dTMP production through pyrimidine salvage pathway using thymidine and thymine supplementation.

A Intracellular dTMP levels in WT and mutant strains upon addition of 1 mM thymine or thymidine to the growth medium. Values are relative to those in WT strain (without any metabolite addition) after 4 h of growth. Mutants show improvement in dTMP levels only upon thymidine addition.

B Intracellular thymine levels in WT and mutant strains increase when grown in the presence of 1 mM thymine in the medium, indicating that it is up taken by the cells.

C Intracellular thymine levels in WT and mutant cells following growth with thymidine supplementation. Increase in thymine levels indicates substantial degradation of thymidine in the salvage pathway through DeoA enzyme.

D Intracellular 2-deoxy-ribose-1-phosphate and 5-phosphate levels in WT and mutant cells. Mutants accumulate substantially high levels of the 5-phosphate variant, indicating its channeling into energy metabolism.

Data information: For all panels, error bars represent SEM of at least three biological replicates.

© 2021 The Authors

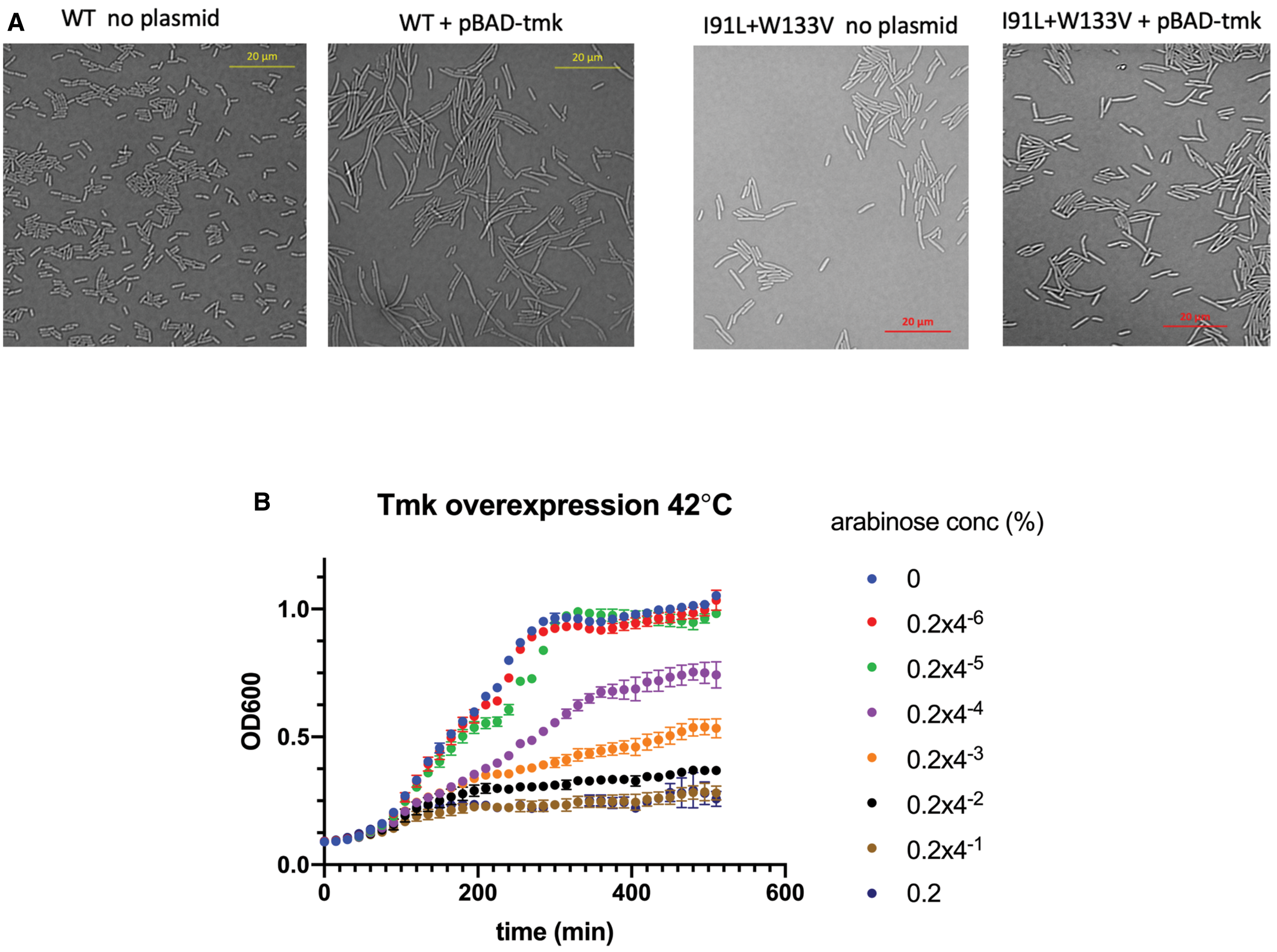

Figure EV5. Effect of overexpression of Tmk on growth and morphology of WT and mutant DHFR strains.

A DIC images of untransformed WT and I91L+W133V mutant cells as well as those transformed with pBAD plasmid that expresses thymidylate kinase under control of arabinose promoter. Cells were grown at 42°C for 4 h (40°C for mutant) in amino acid supplemented M9 medium in the presence of 0.2% of arabinose. While expression of Tmk does not rescue filamentation of mutant cells, it produces filamentation of WT cells.

B Growth curves of WT *E. coli* cells (BW27783) at 42°C following overexpression of Tmk from a pBAD plasmid with different concentrations of arabinose inducer. The data show that overexpression of Tmk is toxic. Error bars represent SD of three technical replicates.

 © 2021 The Authors

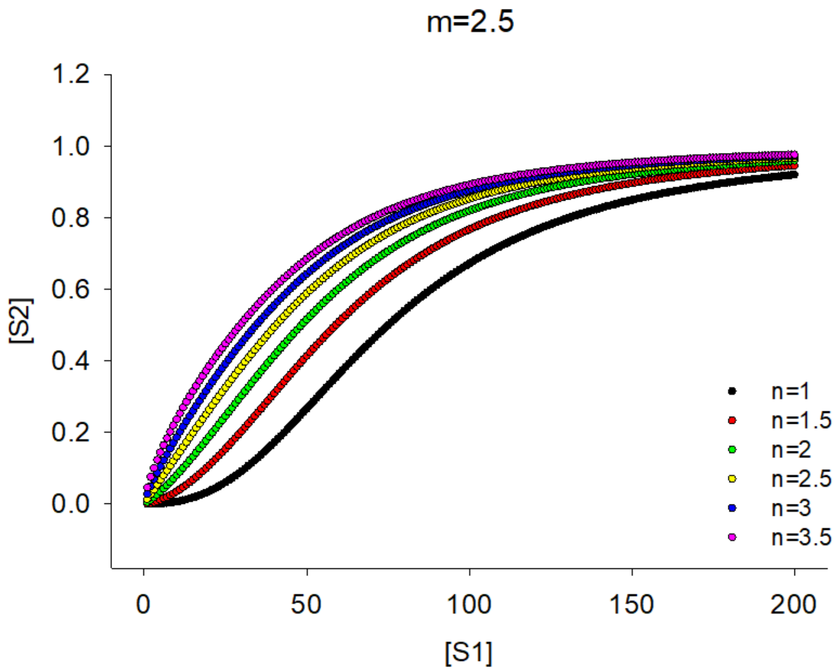

Figure EV6. Steady state concentrations of substrate and product for sequential enzymes and varying values of their Hill coefficients.

Numerical plot of equation 20 showing steady-state concentrations of a product and substrate in an enzymatic chain where sequential enzymes have hill coefficients m and n.

© 2021 The Authors
